# The epidemiology of self-harm in a UK-wide primary care patient cohort, 2001–2013

**DOI:** 10.1186/s12888-016-0753-5

**Published:** 2016-02-29

**Authors:** Matthew J. Carr, Darren M. Ashcroft, Evangelos Kontopantelis, Yvonne Awenat, Jayne Cooper, Carolyn Chew-Graham, Nav Kapur, Roger T. Webb

**Affiliations:** Centre for Mental Health and Safety, Institute of Brain, Behaviour and Mental Health, University of Manchester, Manchester, M13 9PL UK; Centre for Pharmacoepidemiology and Drug Safety, Manchester Pharmacy School, University of Manchester, Manchester, UK; NIHR Greater Manchester Primary Care Patient Safety Translational Research Centre, Manchester, UK; Centre for Health Informatics, Institute of Population Health, University of Manchester, UK, Manchester, UK; NIHR School for Primary Care Research, University of Manchester, Manchester, UK; School of Psychological Sciences, University of Manchester, Manchester, UK; Research Institute of Primary Care and Health Sciences, Keele University, Keele, UK; Manchester Mental Health and Social Care Trust, Manchester, UK

**Keywords:** Self-harm, Attempted suicide, Primary health care, Socioeconomic status, Epidemiology

## Abstract

**Background:**

Most of the research conducted on people who harm themselves has been undertaken in secondary healthcare settings. Little is known about the frequency of self-harm in primary care patient populations. This is the first study to describe the epidemiology of self-harm presentations to primary care using broadly representative national data from across the United Kingdom (UK).

**Methods:**

Using the Clinical Practice Research Datalink (CPRD), we calculated directly standardised rates of incidence and annual presentation during 2001–2013. Rates were compared by gender and age and across the nations of the UK, and also by degree of socioeconomic deprivation measured ecologically at general practice level.

**Results:**

We found significantly elevated rates in females vs. males for incidence (rate ratio - RR, 1.45, 95 % confidence interval - CI, 1.42-1.47) and for annual presentation (RR 1.56, CI 1.54–1.58). An increasing trend over time in incidence was apparent for males (*P* < 0.001) but not females (*P* = 0.08), and both genders exhibited rising temporal trends in presentation rates (*P* < 0.001). We observed a decreasing gradient of risk with increasing age and markedly elevated risk for females in the youngest age group (aged 15–24 years vs. all other females: RR 3.75, CI 3.67–3.83). Increasing presentation rates over time were observed for males across all age bands (*P* < 0.001). We found higher rates when comparing Northern Ireland, Scotland, and Wales with England, and increasing rates of presentation over time for all four nations. We also observed higher rates with increasing levels of deprivation - most vs. least deprived male patients: RR 2.17, CI 2.10–2.25.

**Conclusions:**

Incorporating data from primary care yields a more comprehensive quantification of the health burden of self-harm. These novel findings may be useful in informing public health programmes and the targeting of high-risk groups toward the ultimate goal of lowering risk of self-harm repetition and premature death in this population.

## Background

An estimated 220,000 emergency department presentations of self-harm occur annually in England [1], which places considerable pressure on hospital services. However, because self-harm is the strongest risk factor for subsequent suicide [2], these episodes also represent an important opportunity for prevention [3]. Self-harm also places a heavy burden on primary care services. Thus, guidelines from the National Institute for Health and Care Excellence (NICE) on self-harm management have emphasised the important role played by primary care, yet the majority of research conducted on self-harm has been undertaken in secondary healthcare settings [4, 5]. Over 98 % of the United Kingdom (UK) population is registered with a general practice. Therefore, for most people, their general practitioner (GP) is the first port of call for help, but little is known about self-harm among primary care patients in terms of its frequency of occurrence and associated impact on services.

An assessment of temporal trends and relative risks is needed to direct interventional strategies and allocate resources for effective and efficient primary care service provision. Quantifying differences between demographic subgroups is an important step toward reducing the risk of repetition by ensuring that patients receive adequate levels of assessment, monitoring and therapeutic care. Evidence from secondary care suggests that a significant gender difference exists, with males being at higher suicide risk but with females having a greater incidence of nonfatal self-harm [6, 7]. We anticipated that these findings would be replicated in a primary care cohort, and that we would also observe varying patterns by age, with higher risk expected for the younger age groups [6]. We also expected to find heterogeneity among the constituent nations of the UK (England, Northern Ireland, Scotland, and Wales), as found in relation to death by suicide across the UK [8], and an association with increasing socioeconomic deprivation [9, 10]. In this paper we describe patterns of self-harm risk using directly standardised estimates of incidence and annual presentation rates at general practice.

It was the variation in rates between these subgroups that was our primary focus. Thus, our purpose was not to compare our observed rates against previously published rates based on emergency department presentations. It is currently not possible to conduct a study of self-harm in the UK that captures routinely collected information on all of the following subgroups of people who have harmed themselves: (i) treated in an emergency department with or without subsequent admission; (ii) admitted directly to a general hospital or psychiatric ward without passing through the emergency department; (iii) episode identified by a GP without any preceding secondary care contact. The Clinical Practice Research Datalink (CPRD) did, however, provide a unique opportunity to map out the descriptive epidemiology of self-harm among primary care patients using broadly representative national data sampled from across the whole of the UK population.

## Methods

### Description of the data source

This study was conducted using routinely collected data from the CPRD, the world’s largest population-based, longitudinal, primary care database. This data source contains anonymised patient information entered by general practice staff. Most clinical data is coded using the Read code system [11] and the database includes information on diagnoses, demographics, laboratory tests, medications, and referrals to other healthcare settings. Our study utilised information from 677 general practices, with 10,396,605 patients contributing data at some stage during years 2001–2013, inclusive. We restricted our analyses to patients identified to be of an acceptable quality for research, and registered with a general practice deemed to be ‘up to standard’ on continuity of provision and data completeness criteria. We utilised an ‘open’ cohort study design in that each patient’s time at risk commenced at a different time point, and some exited prior to the end of the study period due to migration, death, or cessation of their practice’s contribution to the CPRD.

### Case definition and clinical coding

In the UK, the National Institute for Health and Clinical Excellence (NICE) guidelines (CG16) define self-harm as any act of “self-poisoning or self-injury, irrespective of the apparent purpose” [4]. Using this broad conceptualisation, we developed a list of Read codes incorporating all cases across the spectrum from milder forms of non-suicidal behaviour through to near-fatal suicide attempts. Initially we searched for all Read codes that included the following terms in their description: ‘deliberate’, ‘intentional’ or ‘self’ (to identify episodes of self-harm/harming, self-injury/injurious behaviour, self-inflicted harm/injury, harm/injury to self, self-poisoning, deliberate overdose, intentional overdose) and ‘suicide attempt’, ‘attempted suicide’ or ‘parasuicide’ (to identify suicide attempts). These candidate codes were then subjected to rigorous clinical review by two expert clinicians within the research team (NK and JC), with any non-relevant codes omitted. Additional potentially relevant codes were identified using the hierarchical structure of the coding system. We excluded codes that referred only to thoughts of self-harm or suicidal ideation and alcohol-related codes, unless intent to actively harm oneself was specified. The resulting list was similar to that used in a recent CPRD validation study [6] and can be accessed at https://clinicalcodes.rss.mhs.man.ac.uk/ [12].

### Self-harm frequency measures

Calculating a rate based on the total number of general practice consultations could be dominated unduly by frequent attenders. We therefore deployed a dual approach at the patient-level to investigate: (i) rates of new cases in the population (incidence); (ii) the proportion of patients affected annually (presentation rates). We restricted our study to this more recent time period to maximise data quality and the relevance of our findings. Patients were eligible for inclusion in a given year if they were aged 15–64 years and registered with a CPRD-contributing practice at the start of the year. The rationale for imposing these age restrictions was that the determinants and implications of self-harm in children and older adults are quite distinct from those of the rest of the population, and therefore warrant separate investigation and consideration. Among older persons who harm themselves, specific mechanisms such as bereavement, loneliness and social isolation [13, 14] and physical illness, multi-morbidity and impairment [14] play a predominant role; children aged below 15 years who harm themselves tend to have an unusually low suicidal intent, and this behaviour is associated with a relatively low long-term risk of suicide [15].

#### Incidence

When calculating incidence rates, denominator estimates were restricted to patients registered at the start of the year with a practice that contributed data throughout the year. Patients with a history of self-harm were excluded. The numerators were estimated as the number of patients included in the denominator with a first recorded episode of self-harm during the year. We excluded patients who were no longer registered on the episode date.

#### Annual presentation rates

Denominator estimates were restricted to patients registered at the start of the year with a practice that contributed data throughout the year. No restriction was placed regarding prior episodes of self-harm. The numerators were estimated as the number included in the denominator, with one or more presentations of self-harm during the year, and still registered on the first of those presentations.

Gender-specific estimates of incidence and annual presentation rates are presented throughout. We report stratified analyses by age (10-year bands), nation (England, Northern Ireland, Scotland, and Wales), and socio-economic deprivation quintiles from 1 (least) to 5 (most deprived).

### Measurement of deprivation

Deprivation quintiles were applied according to the postcode of the general practice of registration, and were derived using the Index of Multiple Deprivation (IMD) for 2010 in each of the four UK nations. The specification differs slightly for each nation [16–19], but throughout the UK the IMD is a measure of area-level deprivation constructed from domains including income, employment, health, education, barriers to services (including housing), crime, and general living environment. In England [16], Wales [18] and Northern Ireland [19], the indices are derived for geographical areas designated as Lower-layer Super Output Areas (LSOA’s), which contain 1000–3000 people and are Census-derived [20]. In Scotland, the small area concentrations are called datazones [17]. The IMD provides a means of ranking and assessing whether an area is more/less deprived than others in the same nation.

### Statistical analyses

The focus of our investigation was comparison of self-harm risk between subgroups of the UK population. Firstly, we compared rates for male versus female patients. Secondly, we conducted gender-specific comparisons across age bands, nations, and deprivation quintiles. Crude rate comparisons could have been misleading if the populations being compared had differed significantly with respect to potential confounders. Therefore, to enhance comparability, all subgroup rates were directly standardised [21] for age, geographical region, and deprivation quintile. We derived directly standardised rates by applying category-specific rates from each subgroup to the demographic distribution of the total CPRD population to produce group-specific rates that would have been observed if the subgroups all had the same distribution. Rates per 10,000 patients are reported as 3-year moving averages, centred on the middle year of each 3-year period.

Variations between demographic subgroups were examined formally using Mantel-Haenszel risk ratios, stratified by study year. Chi-squared tests were applied to assess homogeneity of rates over time and logistic regression to test for temporal linear trends. Each test for trend involved fitting two regression models with time represented as a categorical variable in the first and as a continuous variable in the second. Linear trends were confirmed if a likelihood ratio test on the two models was non-significant but the effect of time was significant in the second model. Significance was assessed at an *α* level of 0.05 (two-sided). All of the analyses were conducted using Stata version 13 [22].

## Results

### Age and gender patterns

**Fig. 1:**
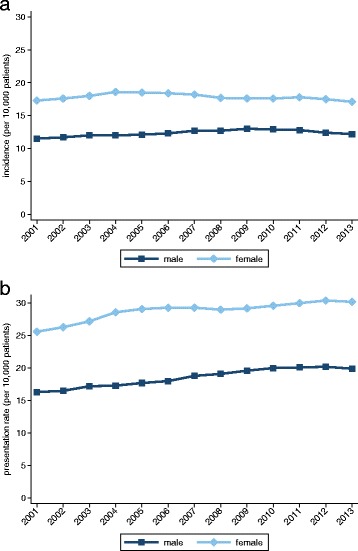
Overall incidence and annual presentation rates. **a** Incidence. **b** Annual presentation rates

We identified 114,323 self-harm episodes in the study cohort of which 94,002 (82.2 %) were self-poisonings, 7,893 (6.9 %) were self-injuries that involved a specified method other than poisoning and 12,428 (10.9 %) were cases with an unspecified method. The directly standardised annual incidence rates were 12.3 and 17.9 per 10,000 male and female patients, respectively. The estimated annual rates of presentation were 18.5 for males and 28.9 for females. Over time, the rates were consistently higher for female patients (Fig. 1). Formal comparisons produced the following female versus male rate ratios: 1.45 (CI: 1.42–1.47) for incident episodes, and 1.56 (CI 1.54–1.58) for all presentations to general practice (Table 1). Although the annual estimates of incidence were heterogeneous through the study period for both genders (males: χ^2^_12_ = 46.1, *P* < 0.001; females: χ^2^_12_ = 48.3, *P* < 0.001), an increasing temporal trend was only observed for males (*P* < 0.001; females: *P* = 0.08). However, both genders displayed increasing linear trends of presentation (*P* < 0.001). The observed gender differences were, to a substantial degree, attributable to the youngest age group: 15–24 years. When omitting this group, the rate ratios fell substantially to 1.20 (CI 1.17–1.22) for incidence and to 1.37 (CI 1.35-1.39) for presentations.

**Table 1:** Risk ratios by gender, age, nation of the UK, and deprivation quintile

	Male			Female		
	Rate/10 k patients^a^	Risk ratio (95 % CI)^b^		Rate/10 k patients^a^	Risk ratio (95 % CI)^b^	
Incident events						
Gender	12.3	-	-	17.9	1.45	(1.42–1.47)
Age:						
15–24	22.9	-	-	45.4	-	-
25–34	15.1	0.66	(0.64–0.69)	16.7	0.37	(0.36–0.38)
35–44	11.8	0.52	(0.50–0.54)	15.3	0.34	(0.33–0.35)
45–54	8.3	0.36	(0.35–0.38)	10.6	0.23	(0.23–0.24)
55–64	5.0	0.22	(0.21–0.23)	5.2	0.11	(0.11–0.12)
Nation:						
England	11.4	-	-	16.8	-	-
N. Ireland	20.1	1.76	(1.66–1.86)	23.6	1.40	(1.33–1.48)
Scotland	16.0	1.40	(1.34–1.45)	22.2	1.32	(1.28–1.37)
Wales	15.1	1.31	(1.26–1.37)	20.5	1.22	(1.18–1.27)
Deprivation:						
1 (least)	8.4	-	-	13.4	-	-
2	10.4	1.23	(1.17–1.29)	16.4	1.22	(1.17–1.27)
3	11.8	1.40	(1.33–1.46)	17.2	1.28	(1.23–1.33)
4	13.7	1.62	(1.55–1.70)	19.0	1.41	(1.36–1.46)
5 (most)	16.7	1.98	(1.90–2.07)	21.9	1.63	(1.57–1.69)
Annual presentations						
Gender	18.5	-	-	28.9	1.56	(1.54–1.58)
Age:						
15–24	28.3	-	-	59.3	-	-
25–34	23.7	0.84	(0.82–0.87)	28.6	0.48	(0.47–0.49)
35–44	20.1	0.71	(0.69–0.74)	28.7	0.49	(0.47–0.50)
45–54	13.9	0.49	(0.47–0.51)	21.9	0.37	(0.36–0.38)
55–64	7.8	0.28	(0.27–0.29)	9.6	0.16	(0.16–0.17)
Nation:						
England	16.9	-	-	26.6	-	-
N. Ireland	33.2	1.96	(1.87–2.05)	42.7	1.60	(1.54–1.66)
Scotland	25.6	1.50	(1.45–1.54)	39.5	1.47	(1.44–1.51)
Wales	22.4	1.31	(1.27–1.36)	32.3	1.21	(1.17–1.24)
Deprivation:						
1 (least)	12.0	-	-	20.8	-	-
2	15.4	1.28	(1.23–1.33)	26.2	1.26	(1.22–1.30)
3	17.6	1.46	(1.41–1.52)	27.3	1.32	(1.28–1.36)
4	20.8	1.73	(1.67–1.80)	31.1	1.50	(1.46–1.54)
5 (most)	26.1	2.17	(2.10–2.25)	37.1	1.79	(1.74–1.84)

*CI* confidence interval

^a^Rates standardised by age band, geographical region and deprivation quintile

^b^Mantel-Haenszel risk ratios, stratified by study year

**Fig. 2:**
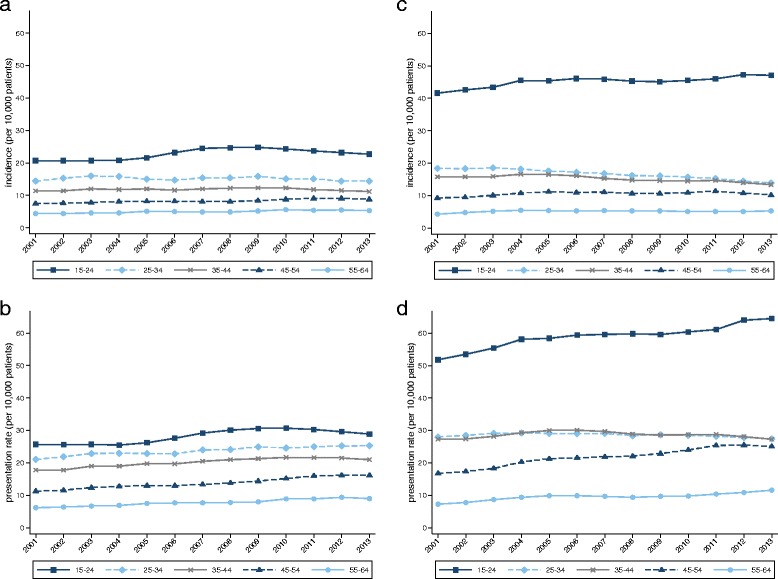
Incidence and annual presentation rates by age band. **a** Incidence in male patients by age band. **b** Annual presentation rates in male patients by age band. **c** Incidence in female patients by age band. **d** Annual presentation rates in female patients by age band

Age-specific rates are illustrated in Fig. 2. Both genders exhibited a decreasing gradient of risk with increasing age. This is reflected in the risk ratios (Table 1; the youngest age group is the reference category). Significant temporal trends in rising incidence were found for males in the youngest (15–24 years) and two oldest age groups (45–54 and 55–64 years). Temporal trends in increasing presentation rates were observed for males in all age groups (*P* < 0.001), and for female patients in all but the second (25–34 years: χ^2^_12_ = 13.5, *P* = 0.33) and third (35–44 years: *P* = 0.90) youngest age bands. The most notable age-gender finding was markedly elevated risk among the youngest females. Comparing females aged 15–24 years with all other females, we observed rate ratios of 3.75 (CI 3.67–3.83) for incidence and 2.62 (CI 2.57–2.66) for annual presentation.

### Variation across the UK nations and by deprivation level

**Fig. 3:**
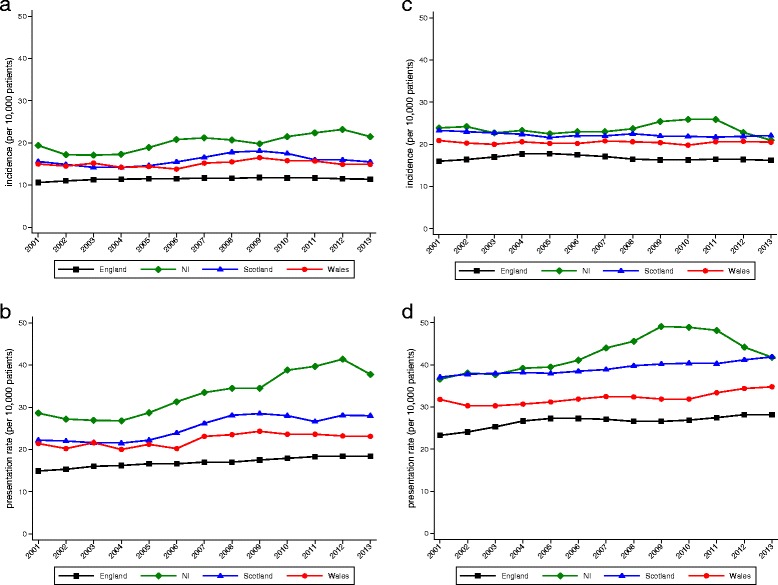
Incidence and annual presentation rates by nation of the UK. **a** Incidence in male patients by nation. **b** Annual presentation rates in male patients by nation. **c** Incidence in female patients by nation. **d** Annual presentation rates in female patients by nation

Figure 3 presents rates of self-harm stratified by UK nation. Northern Ireland had the highest rates of incidence and annual presentation, followed by Scotland then Wales. Both rates were consistently much lower in England (Table 1). Linear temporal trends in rising incidence were found for males of all nationalities, but no significant trends were discernible for females. We did however find steadily increasing rates of presentation for both genders across all nations, particularly in Northern Ireland where rates began increasing substantially in 2004. These increases continued until 2009 for females and 2012 for males.

**Fig. 4:**
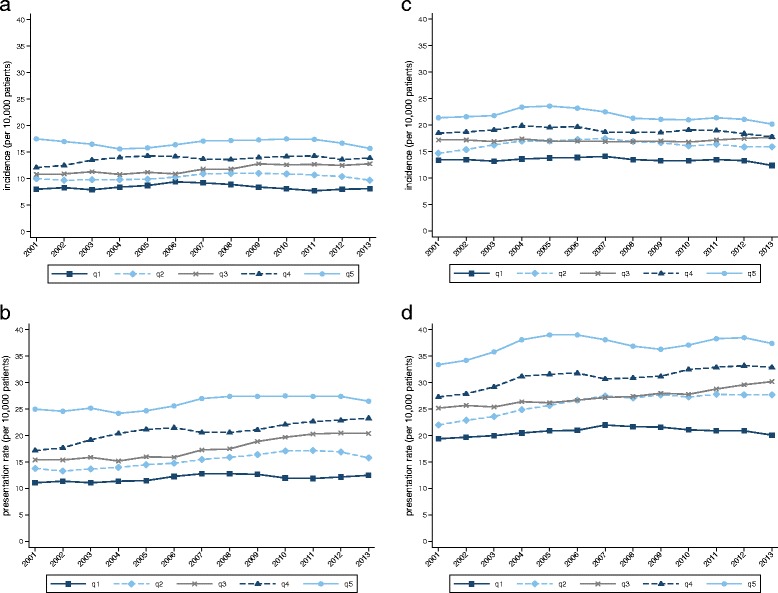
Incidence and annual presentation rates by deprivation quintile. **a** Incidence in male patients by deprivation quintile. **b** Annual presentation rates in male patients by deprivation quintile. **c** Incidence in female patients by deprivation quintile. **d** Annual presentation rates in female patients by deprivation quintile

Figure 4 illustrates rates stratified by deprivation quintile. Significant increases in presentation rates over time were found across all quintiles for both genders. A strong association between increasing levels of deprivation and risk is clearly visible (Fig. 4, Table 1), for incidence and annual presentation rates. Comparing incidence among patients in the two most deprived quintiles with those in the two least deprived quintiles, we observed risk ratios of 1.61 (CI 1.56–1.66) for males and 1.36 (CI 1.32–1.39) for females. Similarly, the annual presentation risk ratios were 1.70 (CI 1.66–1.74) and 1.44 (CI 1.42–1.47), respectively. However, the increase in risk was not truly linear across all five quintiles, with greater incremental increases observed at the extremes: i.e. the increases in risk between quintiles 1 to 2 (least deprived) and quintiles 4 to 5 (most deprived) were greater than between quintiles 2 to 3 and 3 to 4.

**Fig. 5:**
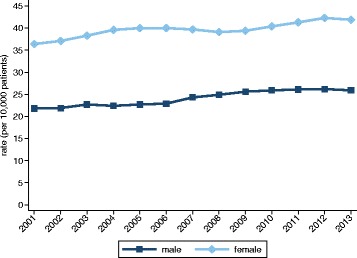
Rates of all presentations

The focus of this paper is at the patient level and we have presented annual rates that represent the proportion of individuals affected on an annual basis. Our presentation rates are thus derived from binary patient-level data. We also conducted a sensitivity analysis on our rate specification and modelled the total number of presentations, or ‘contact days’, as count data; i.e. patients could be counted more than once in a given year. In Fig. 5 we present the rates of overall presentation by gender. We found similar relative relationships between genders and other subgroups of the population using either definition of presentation rates.

## Discussion

### Summary of findings

This study provides new epidemiological evidence on the presentation of self-harm to general practice, comparing demographic subgroups using a large primary care database. Rates of gender-specific incidence and presentation were generally increasing over the duration of the study with consistently higher rates for females. Self-harm incidence and presentation rates decreased incrementally with age. Substantially higher rates were observed for females aged 15–24 years, and this group dominated the overall gender differences. We also found lower rates in England when compared against the other nations of the UK, and a gradient of increasing risk across deprivation quintiles (from least to greatest level of deprivation) with a notable increase in risk for the most deprived quintile. This suggests that the psychosocial determinants of self-harming behaviour cluster strongly in the poorest localities.

### Comparison with existing evidence

The term ‘self-harm’ encompasses a range of methods with varying degrees of intent [9]. Most of the evidence on self-harm and attempted suicide has arisen solely through data collected in hospital settings. There is a dearth of evidence in a primary care context in the UK and other countries. This lack of attention, and the limited instruction issued to GPs, is highlighted in recent NICE guidelines on the longer term management of self-harm [5]. The guideline report includes a section that highlights the importance of primary care in managing the problem, but only three out of a total of 57 recommendations relate specifically to this healthcare tier.

We found self-harm rates to be generally increasing over our 13-year study period. This is consistent with findings from the previous decade (1990–1999), and represents a continuation of existing trends [7]. A rise in both sexes reporting self-harm as a response to employment and financial problems has been reported in recent years [23]. We found significantly higher rates for female patients and the younger age groups. Again, these findings are consistent with the evidence from secondary care settings [1, 7]. Differences in method selection may play a key role with men tending to use more violent methods when engaging in suicidal behaviour, and are therefore more likely to die as a result [24]. This may explain the higher rate of nonfatal self-harm observed in women. Men also more frequently express their psychological distress by alcohol misuse [25] and aggression or violence towards others [26].

When examining rates across the UK, we found lower rates in England compared to Northern Ireland, Scotland and Wales. These results mirror findings for completed suicide, where large disparities have been found between national rates [8, 10]. Notably, it has been observed that rates in Scotland have increased markedly relative to those in England in recent decades [27]. Employment and socio-economic status have been discussed as risk factors for self-harm in many studies [7, 9, 10]. These factors, along with mental illness and alcohol misuse, could explain the heterogeneity in self-harm rates we observed across the UK nations [28].

The need for closer relationships between primary and secondary healthcare practitioners, and between practices and community mental health teams, has also been emphasised [29, 30]. In 2002, it was reported that the UK has one of the highest rates of self-harm in Europe, at 40 per 10,000 population [31]. However, due to a lack of national databases for self-harm, reliable figures on the frequency of occurrence are not readily available. A multi-centre study of emergency department presentations of self-harm in three cities across England gave rates during 2000–2007 of 36, 46 and 44 per 10,000 population in Oxford, Manchester and Derby respectively [7]. The corresponding gender-specific rates were 31, 37 and 37 for males and 41, 54 and 51 for females. Differences in reported rates of self-harm between primary and secondary care may result from the fact that only around half of patients visit their GP in the month following a presentation to a hospital [32], and practices are only notified of an episode in approximately half of cases involving a mental health specialist [33]. It is also likely that some patients only present to general practice settings and not hospital services.

### Strengths and limitations

Our study’s main strength was the scale and scope of the CPRD, which enabled us to examine an outcome that is comparatively rare in the general population. A comprehensive self-harm database does not currently exist in the UK, but this is true of all other countries worldwide, except for the Republic of Ireland, which has established a national registry of hospital presentations [34]. Multicentre monitoring of secondary care presentations has been undertaken, with the purpose of characterising the epidemiology of self-harm at a population level beyond reports from single centres [1]. While these studies provide useful data, they report findings from just three cities and therefore do not provide a comprehensive national picture. Hospital Episodes Statistics (HES) linked to national mortality records have recently been used to examine self-harm in England [35]. However, this dataset captures only the more medically serious cases that require admission. The general practices that contribute to the CPRD provide a broadly representative UK-wide sample with overall distributions of age and gender corresponding to those of the whole population. Because of this national representativeness, it is unlikely that any age or gender bias will have entered our analyses. Similar relative distributions have been observed in other studies [7].

On an annual basis, we investigated incident episodes and the proportion of patients presenting to general practice on at least one occasion. This is a new definition for primary care in the context of a stable, non-transient, subset of the population. Thus, by placing practice registration restrictions, we ensured reliable comparisons between subgroups with an emphasis on temporal trends and relative risks. Internally standardised rate estimates have been used throughout. However, due to the nationally representative nature of the data, we found that crude and standardised estimates were almost identical.

Our study had several limitations. Firstly, comprehensive ascertainment of all self-harm episodes among this primary care patient cohort was reliant on patient disclosure of self-harming behaviour to their GPs, the consistency of clinical coding practice among GPs, and the completeness of notification of other cases by hospital emergency departments. Validation of self-harm case definition, by chart review of medical records, is not possible when using data from the CPRD. It is also possibile that increasing awareness of self-harm introduced a degree of surveillance bias into our analyses of temporal trends.

Secondly, it was necessary to restrict our analysis of incident episodes to the identification of first recorded events. As we did not have any means of ensuring that these were genuine first episodes, it is possible that some patients will have previously self-harmed, perhaps prior to their current practice registration. Finally, research has shown that self-harm may be more strongly related to individual socioeconomic factors than to geographical area characteristics [36]. As this patient-level data was unavailable, it was a necessary limitation to use an ecological small area-level measure of deprivation as a proxy.

## Conclusions

Our findings highlight subgroups of the GP registered primary care patient population with an elevated risk of self-harm. These patterns of risk have not previously been illustrated in a nationally representative dataset in the UK. As self-harm is the main risk factor for subsequent suicide [2], GPs are likely to play a key role in the provision of care following an episode of self-harm [37]. However, very little is known about self-harming patients in the context of general practice. Our findings could inform the development of guidelines for improved management of patients with a propensity for self-harm. Further research is needed to investigate mental illness diagnoses, treatment and GP referrals to specialist services, risks of repetition, suicide and other causes of premature death, and how risks might be lowered through effective intervention.

### Ethics approval

The study was approved as being both ethically and scientifically sound by the independent scientific advisory committee (ISAC) for research using the Clinical Practice Research Datalink (CPRD): Reference number: 13_122ARA2.

### Availability of data

Clinical Practice Research Datalink (CPRD) study datasets cannot be shared due to licencing restrictions. Researchers wishing to conduct their own investigations using CPRD data should contact the Knowledge Centre directly at the following email address: kc@cprd.com. All the clinical code-lists used in the analysis of CPRD data in this study are available at https://clinicalcodes.rss.mhs.man.ac.uk/. The full Stata code used for the analysis of CPRD data is available from the authors.

## Abbreviations

CI: confidence interval
CPRD: clinical practice research datalink
GP: general practitioner
IMD: index of multiple deprivation
LSOA: lower-layer super output area
NICE: National Institute for Health and Clinical Excellence
RR: rate ratio
UK: United Kingdom

## References

[1] Hawton K, Bergen H, Casey D, Simkin S, Palmer B, Cooper J (2007). Self-harm in England: a tale of three cities. Multicentre study of self-harm. Soc Psychiatry Psychiatr Epidemiol.

[2] Cooper J, Kapur N, Webb R, Lawlor M, Guthrie E, Mackway-Jones K (2005). Suicide after deliberate self-harm: a 4-year cohort study. Am J Psychiatry.

[3] Kapur N (2009). Health services and suicide prevention. J Ment Health.

[4] National Institute for Clinical Evidence. Self-harm: the short-term physical and psychological management and secondary prevention of self-harm in primary and secondary care. NICE Guideline CG16; 2004. https://www.nice.org.uk/guidance/cg16. Accessed 23 June 2015.

[5] National Institute for Health and Clinical Excellence. Self-harm: longer-term management. NICE Guideline CG133. 2011. https://www.nice.org.uk/guidance/cg133. Accessed 23 June 2015.

[6] Thomas KH, Davies N, Metcalfe C, Windmeijer F, Martin RM, Gunnell D (2013). Validation of suicide and self-harm records in the Clinical Practice Research Datalink. Br J Clin Pharmacol.

[7] Bergen H, Hawton K, Waters K, Cooper J, Kapur N (2010). Epidemiology and trends in non-fatal self harm in three centres in England: 2000–2007. Br J Psychiatry.

[8] The National Confidential Inquiry into Suicide and Homicide by People with Mental Illness (2014). Annual Report: England, Northern Ireland, Scotland and Wales - July 2014.

[9] Skegg K (2005). Self-harm. Lancet.

[10] Brock A, Baker A, Griffiths C, Jackson G, Fegan G, Marshall D (2006). Suicide trends and geographical variations in the United Kingdom, 1991–2004. Health Stat Q.

[11] Health and Social Care Information Centre. NHS UK Read Codes. https://isd.hscic.gov.uk/trud3/user/guest/group/0/pack/9. Accessed 23 June 2015.

[12] Springate DA, Kontopantelis E, Ashcroft DM, Olier I, Parisi R, Chamapiwa E (2014). ClinicalCodes: an online clinical codes repository to improve the validity and reproducibility of research using electronic medical records. PLoS ONE.

[13] De Leo D, Padoani W, Scocco P, Lie D, Bille-Brahe U, Arensman E (2001). Attempted and completed suicide in older subjects: results from the WHO/EURO multicentre study of suicidal behaviour. Int J Geriatr Psychiatry.

[14] Lebret S, Perret-Vaille E, Mulliez A, Gerbaud L, Jalenques I (2006). Elderly suicide attempters: characteristics and outcome. Int J Geriatr Psychiatry.

[15] Hawton K, Harriss L (2008). Deliberate self-harm by under 15-year-olds: characteristics, trends and outcome. J Child Psychol Psychiatry.

[16] Communities and Local Government (2011). The English indices of deprivation 2010: technical report.

[17] The Scottish Government. Scottish Index of Multiple Deprivation (SIMD). http://simd.scotland.gov.uk/publication-2012/. Accessed 23 June 2015.

[18] The Welsh Government. Welsh Index of Multiple Deprivation (WIMD). http://gov.wales/statistics-and-research/welsh-index-multiple-deprivation/?lang=en. Accessed 23 June 2015.

[19] Northern Ireland Statistics and Research Agency (NISRA). Northern Ireland Multiple Deprivation Measure 2010. http://www.nisra.gov.uk/deprivation/nimdm_2010.htm. Accessed 23 June 2015.

[20] Office for National Statistics: Super Output Area (SOA). http://www.ons.gov.uk/ons/guide-method/geography/beginner-s-guide/census/super-output-areas--soas-/index.html. Accessed 23 June 2015.

[21] Hennekens CH, Buring JE (1987). Epidemiology in Medicine.

[22] StataCorp (2013). Stata Statistical Software: Release 13.

[23] Dickson S, Steeg S, Gordon M, Donaldson I, Matthews V, Kapur N (2011). The Manchester Self-Harm Project: Self-Harm in Manchester, January 2008 - December 2009.

[24] Mościcki EK (1994). Gender differences in completed and attempted suicides. Ann Epidemiol.

[25] Markman Geisner I, Larimer ME, Neighbors C (2004). The relationship among alcohol use, related problems, and symptoms of psychological distress: gender as a moderator in a college sample. Addict Behav.

[26] Chen P, Coccaro EF, Jacobsen KC (2012). Hostile attributional bias, negative emotional responding, aggression in adults: moderating effects of gender and impulsivity. Aggress Behav.

[27] Mok PL, Kapur N, Windfuhr K, Leyland AH, Appleby L, Platt S (2012). Trends in national suicide rates for Scotland and for England & Wales, 1960–2008. Br J Psychiatry.

[28] Mok PLH, Leyland AH, Kapur N, Windfuhr K, Appleby L, Platt S (2013). Why does Scotland have a higher suicide rate than England? An area-level investigation of health and social factors. J Epidemiol Community Health.

[29] Chew-Graham CA, Slade M, Montana C, Stewart M, Gask L (2007). A qualitative study of referral to community mental health teams in the UK: exploring the rhetoric and the reality. BMC Health Serv Res.

[30] Chew-Graham CA, Slade M, Montana C, Stewart M, Gask L (2008). The loss of doctor-to-doctor communication: lessons from the reconfiguration of mental health. J Health Serv Res Policy.

[31] Horrocks J, House A (2002). Self-poisoning and self-injury in adults. Clin Med.

[32] Gunnell D, Bennewith O, Peters TJ, Stocks N, Sharp DJ (2002). Do patients who self-harm consult their general practitioner soon after hospital discharge? A cohort study. Soc Psychiatry Psychiatr Epidemiol.

[33] Cooper J, Murphy E, Jordan R, Mackway-Jones K (2008). Communication between secondary and primary care following self-harm: are National Institute of Clinical Excellence (NICE) guidelines being met?. Ann Gen Psychiatry.

[34] Perry IJ, Corcoran P, Fitzgerald AP, Keeley HS, Reulbach U, Arensman E (2012). The incidence and repetition of hospital-treated deliberate self harm: findings from the World’s first national registry. PLoS ONE.

[35] Singhal A, Ross J, Seminog O, Hawton K, Goldacre MJ (2014). Risk of self-harm and suicide in people with specific psychiatric and physical disorders: comparisons between disorders using English national record linkage. J R Soc Med.

[36] Johnston A, Cooper J, Webb R, Kapur N (2006). Individual- and area-level predictors of self-harm repetition. Br J Psychiatry.

[37] Houston K, Haw C, Townsend E, Hawton K (2003). General practitioner contacts with patients before and after deliberate self harm. Br J Gen Pract.

